# Pre-diagnosis Multidisciplinary Tumor Board and Time to Staging in Lung Cancer: The Case Western MetroHealth Experience

**DOI:** 10.7759/cureus.6595

**Published:** 2020-01-08

**Authors:** Prashanth Thalanayar Muthukrishnan, Maya Ratnam, Minh-Tri Nguyen, Michael Le, Douglas Gunzler, Debora Bruno, Michael Infeld

**Affiliations:** 1 Pulmonary and Critical Care Medicine, Case Western Reserve University School of Medicine / Metrohealth Medical Center, Cleveland, USA; 2 Medicine, Case Western Reserve University School of Medicine / Metrohealth Medical Center, Cleveland, USA; 3 Epidemiology and Public Health, Center for Health Care Research and Policy, Metrohealth Medical Center, Cleveland, USA; 4 Oncology, Case Western Reserve University School of Medicine / University Hospitals Medical Center, Cleveland, USA

**Keywords:** multidisciplinary care, multidisciplinary tumor board, pre-diagnosis tumor board, pd-mtb, metrohealth, thoracic oncology, lung cancer, time to staging, time to therapy

## Abstract

Introduction

National guidelines support the discussion of cancer patients by multidisciplinary tumor boards (MTB). We researched whether early pre-diagnosis multidisciplinary tumor board discussions are associated with shorter times to staging in lung cancer.

Methods

We reviewed our institution’s lung cancer and MTB registries to retrospectively study if an early discussion at pre-diagnostic MTB (pd-MTB) influenced the timeliness of diagnostic evaluation. Over 14 months, 161 consecutive patients with a diagnosis of lung cancer were included. Fifty-five patients were presented at pd-MTB while 106 (controls) patients were not. The primary outcome was the difference in the time interval from suspicious imaging (Ix) to completion of staging (Sx). Outcomes were adjusted for key confounders with a multiple regression analysis.

Results

For stages I, II, and III lung cancer, where time to therapy matters, early discussion of patients with nodules suspicious for malignancy at pd-MTB was associated with no time delays when compared to patients who were not discussed in pd-MTB. The mean time intervals for imaging to staging (with standard deviations) are 65 days in controls (sd = 42.67) and 75 days (sd = 58.27) in tumor board cases (p=0.39). Adjusting for confounders with a multiple regression analysis among all stages revealed a similar lack of difference in time intervals to diagnosis, staging, and therapy.

Conclusion

Our stage I-III lung cancer cases (pd-MTB) completed staging in a timely manner, similar to controls (no pd-MTB). The severity of illness at presentation and the availability of diagnostic services and others likely influence the results. Our manuscript shares important numerical data on timelines during cancer diagnosis and treatment. Using this data, prospective registries examining the process workflow may help standardize cancer quality goals and maximize referrals from primary-care/specialty providers. The key findings in our study create a paradigm for future studies to create and achieve “door-to-balloon” time targets for lung cancer care (akin to cardiac care) across different styles of tumor boards.

## Introduction

The U.S. healthcare system has encouraged and assessed the quality of teamwork in recent years. For decades, multidisciplinary tumor boards have exemplified the advantages of cooperation and pooling provider expertise. Several studies have demonstrated an association between multidisciplinary tumor board (MTB) discussion and the quality of cancer care [1]. MTB presentation was associated with greater adherence to treatment and staging guidelines as well as shorter time from diagnosis to treatment [2]. Recent advances in lung cancer evaluation, such as large-scale computed tomography (CT) lung nodule screening, pre-diagnosis positron emission tomography (PET) scanning, endobronchial ultrasound (EBUS) biopsy, and need for mutational analysis of tumors have added to the complexity of identifying the optimal method for diagnostic tissue procurement [3-4]. Endobronchial or CT-guided biopsy prior to the evaluation of individual patient circumstances may not be in the best interest of many patients. Recent studies, such as the ReCAP initiative, have addressed this issue, but there is a paucity of data on whether an early multidisciplinary discussion on the possible lung cancers may improve patient outcomes [5]. A question remains as to what helps us identify the best time to include a case in the tumor board list. In 2015, our institution initiated a specific MTB session to plan the efficient evaluation of chest nodules and masses prior to tissue diagnosis. We performed a retrospective review of consecutive patients diagnosed with lung cancer in 2016 from our cancer clinic and tumor board registry to investigate whether these early discussions affected the time required to complete a lung cancer evaluation.

## Materials and methods

Every other week, an MTB meeting was convened to discuss lung nodules and masses. The discussants included physicians from radiology, medical and radiation oncology, and pulmonary medicine, with experience in EBUS. The same group plus pathology and cardiothoracic surgery representatives conducted a separate tumor board to discuss previously diagnosed lung cancer for planning further steps on alternate weeks.

**Figure 1:**
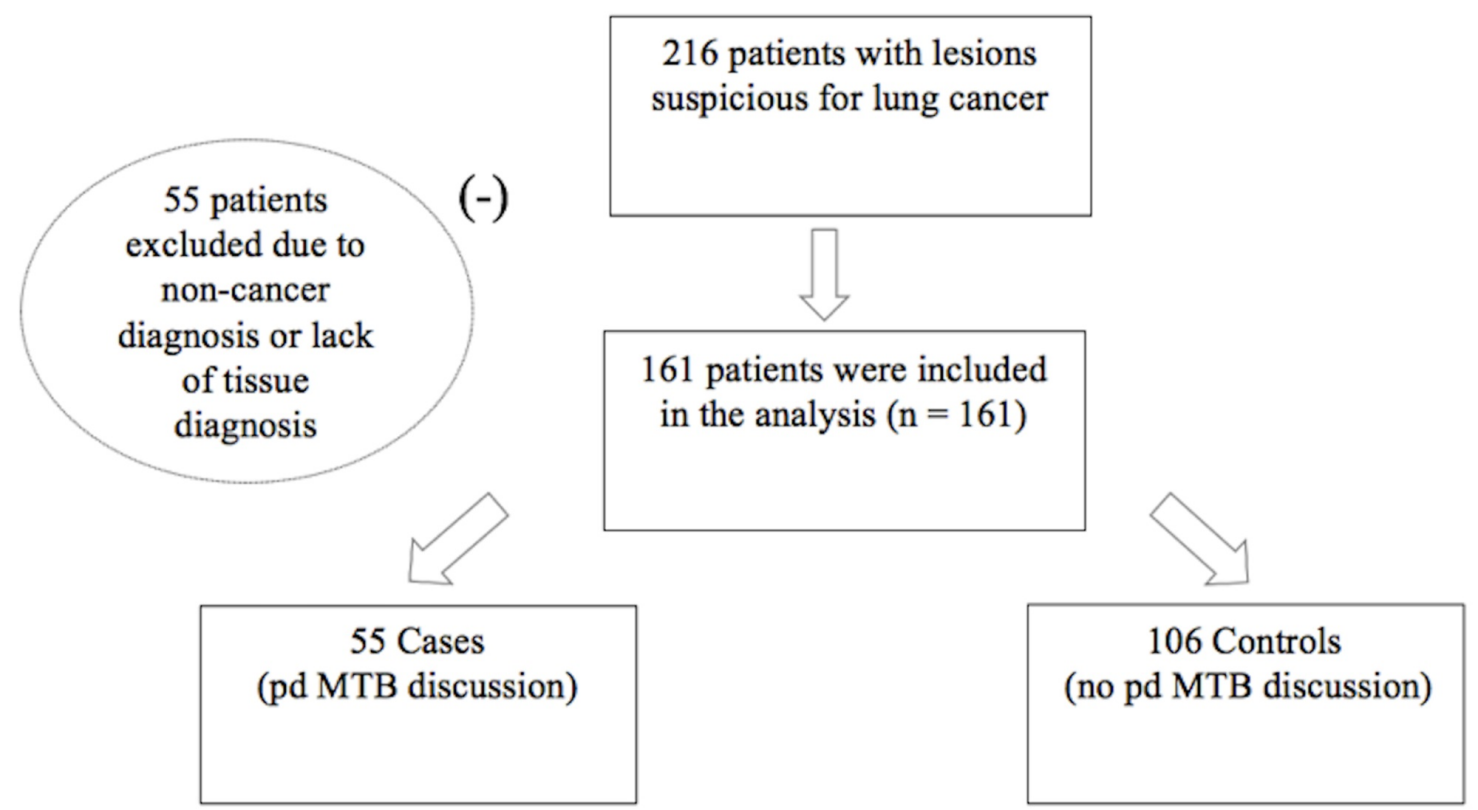
Flowchart depicting the disposition of all cases pd-MTB: pre-diagnosis multidisciplinary tumor board

A total of 216 cases of newly suspected lung cancer were noted in the registries of the lung cancer clinic and tumor board from December 2015 to January 2017. Of these, 55 patients were excluded because they did not have any tissue diagnosis or they had a diagnosis other than cancer. Confirmation of suspected cancer was not pursued in patients with a poor functional status, where the risk of biopsy outweighed potential benefits or if the patient refused invasive testing. The remaining 161 patients with confirmed lung cancer were divided into those who had early tumor board discussions (cases: pre-diagnostic MTB (pd-MTB)) and those who did not (controls: no pd-MTB). Cases presented at MTB for the first time after a tissue diagnosis had already been established were considered controls. A total of 55 cases and 106 controls were evaluated, as shown in Figure 1. This study was conducted in accordance with the amended Declaration of Helsinki. The local institutional review board approved the protocol. Informed consent was waived by the IRB since patient information used for this study was deidentified and no additional patient contact was needed.

Data were collected at the time of registration into the cancer clinic registry, including age, sex, race, insurance coverage, smoking status, symptoms, nodule size, and whether the nodule was discovered as an incidental finding in a study done for another purpose or as part of the CT nodule screening program. Also recorded were whether EBUS was performed, the final cancer stage, and therapy offered. Vital status (alive or dead) was assessed as of July 2017.

Nodules suspicious for malignancy were defined in this study as any lung imaging reporting and data system (LUNG-RADS) 3 or 4 A, B, and X nodules. These include any solid nodule > 8 mm at baseline; any growth in a solid nodule size > 6 mm; sub-solid nodule with solid component > 6 mm or growing/new solid component > 4 mm; or any growth in a ground glass nodule > 6 mm) [6-7]. The date that the imaging was deemed suspicious for cancer (Ix) was defined by the initiation of further evaluation either by ordering a PET-CT scan or pulmonary consultation (whichever was earlier). The date of tissue diagnosis (Dx) was the date when the final pathology report reported lung cancer. The date of staging completion (Sx) was the earliest date when both radiological and pathologic confirmation of the cancer stage was finalized. This included brain imaging when indicated by the National Comprehensive Cancer Network (NCCN) guidelines [8]. The date of pd-MTB note was identified by chart review. Patients with diagnosis, staging, and therapy on the same day via surgical resection of small peripheral nodules had time from diagnosis to staging to therapy as 0 days. The date when initial therapy was instituted (Tx) was the date of surgical resection, initiation of radiation, chemotherapy, palliative, and/or hospice care.

The primary endpoint of this study was the interval between when imaging prompted further investigation for cancer to the completion of cancer staging (Ix to Sx) among all four stages of patients. Secondary variables included time from imaging to diagnosis (Ix to Dx) and treatment (Ix to Tx). The interval for diagnosis to staging (Dx to Sx); diagnosis to treatment (Dx to Tx); and staging to treatment (Sx to Tx) were also analyzed. Because diagnosis and staging are likely to proceed more rapidly in advanced, symptomatic disease typically evaluated in the inpatient setting and where tissue procurement decisions are simpler, we also analyzed the data for stages I-III separately, excluding the half of our sample with stage IV disease. With the above methodology as well as multiple regression analysis, we have attempted to account for the key confounders that would influence both the time taken to complete a staging workup as well as the decision to include in the tumor board.

Statistical methods

Analyses were conducted in R software version 3.4.2 in the R studio environment for Windows. The level of significance was α=0.05 unless noted otherwise. The Mann-Whitney U test, Fisher’s exact test, and Cochran-Armitage trend test were used where appropriate, comparing cases and controls for demographic and independent variables. The Mann-Whitney U test was used to compare primary and secondary outcomes. A multiple regression analysis was conducted to evaluate the association between time from imaging and staging among controls and cases while adjusting for key confounding variables, including stage, cancer type, performance of EBUS, and type of clinical presentation.

## Results

Our low-dose CT cancer-screening program supplied 14% of the patients evaluated. Cases (pd-MTB) were nearly four times as likely as controls to have had their cancer discovered by a screening CT (p=0.01) and about 20% more of the control patients presented with a symptomatic lung mass. Most patients had non-small cell lung cancer (NSCLC) (84% and 90.9% in controls and cases, respectively). Stage IV disease was represented at 52% in the controls compared to 36% of the cases. This mirrors the observation that percentage-wise nearly four times as many patients had mediastinal staging by EBUS among the cases as compared to the controls (p<0.001). There was no significant difference in sex, race, current smoking status, insurance status, nodule size, therapy type, or one-year mortality between the groups. The rate of hospice referral was almost twice as frequent in controls versus cases, with a 10% higher mortality in the control group, but this was not a statistically significant difference. Patient characteristics are summarized in Table 1.

**Table 1:** Patient characteristics EBUS: endobronchial ultrasound; LDCT: low-dose computed tomography Reporting values as (n,%); mean (standard deviation) for continuous measures and number of subjects in each category (percentage of subjects in each category) for discrete measures with p-values from the Mann-Whitney U test, Fisher’s exact test, and Cochran-Armitage trend test, where appropriate

Variable (n,%)	Category	Controls (n/106,%)	Cases (n/55,%)	p-value
Stage	I	26 (24.5)	16 (29.1)	0.14
	II	9 (8.5)	8 (14.5)	
	III	16 (15.1)	11 (20.0)	
	IV	55 (51.9)	20 (36.4)	
Lung cancer type	Non-small cell	89 (84.0)	50 (90.9)	0.33
	Small cell	17 (16.0)	5 (9.1)	
Sex	Female	49 (46.2)	24 (43.6)	0.86
	Male	57 (53.8)	31 (56.4)	
Race	Black	33 (31.1)	16 (29.1)	0.95
	Other	4 (3.8)	2 (3.6)	
	White	69 (65.1)	37 (67.3)	
Smoking	Current smoker	69 (65.1)	41 (74.5)	0.28
	Ex-smoker/ None	37 (34.9)	14 (25.5)	
Vital status	Alive	65 (61.3)	39 (70.9)	0.29
	Dead	41 (38.7)	16 (29.1)	
Clinical presentation	LDCT Screen	8 (7.5)	15 (27.3)	0.01
	Incidental nodule	35 (33.0)	14 (25.5)	
	Symptomatic mass	63 (59.4)	26 (47.3)	
Nodule size (cm)	Mean size (sd)	3.87 (2.86)	3.52 (2.29)	0.73
EBUS bronchoscopy	No	93 (87.7)	29 (52.7)	<0.001
	Yes	13 (12.3)	26 (47.3)	
Insurance	Insured	99 (93.4)	54 (98.2)	0.31
	Not insured	7 (6.6)	1 (1.8)	
Therapy	Hospice	24 (22.6)	7 (12.7)	0.62
	Surgery	18 (17.0)	9 (16.4)	
	Radiation	19 (17.9)	11 (20.0)	
	Chemotherapy	15 (14.2)	10 (18.2)	
	Chemo-Radiation	30 (28.3)	18 (32.7)	

Outcomes of interest

When looking at all comers (stages I-IV), the cases with early tumor board discussion took an average of 70 days to complete staging. This was 21 days longer than the control group as seen in Table 2 (p<0.001). Relative delays were also noted between imaging and diagnosis (61 days versus 37 days, p<0.001), staging and therapy (44 days versus 25 days, p=0.01), and imaging to therapy (103 days versus 74 days, p<0.001).

**Table 2:** Primary and secondary outcomes pd-MTB: pre-diagnostic multidisciplinary tumor board. Mean number of days between imaging, staging, diagnosis, and initiation of therapy. Reporting mean (standard deviation) with p-values from the Mann-Whitney U test. Median values are also available at request

Time intervals	Stages included	Controls (no pd-MTB) Mean (sd)	Cases (pd-MTB) Mean (sd)	p-value
Imaging to Staging	All (n=161)	49.33 (55.04)	70.15 (53.87)	<0.001
	Stages I-III (n=86)	65.45 (42.67)	75.77 (58.27)	0.39
Imaging to Diagnosis	All	37.36 (50.30)	61.71 (54.45)	<0.001
	Stages I-III	54.31 (42.75)	69.71 (59.06)	0.13
Diagnosis to Staging	All	11.97 (21.87)	8.44 (12.86)	0.07
	Stages I-III	11.14 (17.27)	6.06 (11.38)	0.07
Staging to Therapy	All	25.23 (54.25)	44.69 (71.26)	0.01
	Stages I-III	24.24 (30.31)	41.46 (54.64)	0.03
Diagnosis to Therapy	All	37.20 (58.56)	53.13 (72.88)	0.06
	Stages I-III	35.37 (33.96)	47.51 (54.15)	0.28
Imaging to Therapy	All	74.56 (81.34)	114.84 (103.96)	<0.001
	Stages I-III	89.69 (53.49)	117.23 (103.48)	0.15

The statistical significance of the delay was largely eliminated when stage IV patients were excluded from the analysis. Time from imaging to staging (Ix Sx) in patients staged from I to III was a mean of 75 days in cases and 65 days in the control group (p=0.39). There were no significant differences for the secondary outcomes in the sub-group analysis, with the exception of average time from staging to therapy (Sx Tx), which remained significantly delayed - 41 days versus 24 days comparing cases to controls (p=0.03). This variable is independent of the efficiency of lung cancer evaluation, as it occurs after its completion. It is likely related to the type and aggressiveness of therapies employed. Median value data showed similar features (available at the reader’s request).

A multiple regression analysis of the primary outcome failed to show a statistically significant delay when adjusted for stage, pathological type, performance of endobronchial ultrasound, and clinical presentation of cancer, as shown in Table 3.

**Table 3:** Multiple regression analysis for imaging to staging pd-MTB: pre-diagnostic multidisciplinary tumor board; EBUS: endobronchial ultrasound; LDCT: low-dose computed tomography; multiple R-squared = 0.185; adjusted R-squared = 0.142; B: unstandardized beta; SE B: standard error for the unstandardized beta; t: t-test statistic; p: probability value

Coefficient	B	SE B	t	p
(Intercept)	66.97	13.25	5.06	<0.001
pd-MTB	14.51	9.51	1.53	0.129
Stage 1 (Reference)				
Stage 2	-38.31	14.94	-2.56	0.011
Stage 3	-36.33	14.58	-2.49	0.014
Stage 4	-39.41	11.13	-3.54	<0.001
Lung Cancer Type Non-small Cell	-17.26	12.25	-1.41	0.161
Use of EBUS Bronchoscopy	16.52	11.24	1.47	0.144
Presentation: Symptomatic Mass (Reference)				
LDCT Screen	22.04	13.59	1.62	0.107
Incidental Nodule	8.44	13.66	0.62	0.538

## Discussion

Timely diagnosis and staging are important for optimizing cancer care. Accurate staging often includes mediastinal evaluation by endobronchial ultrasound or mediastinoscopy. If diagnosis and staging can be combined into a single procedure, efficiency and cost-effectiveness should improve. Tissue procurement in lung cancer patients can be difficult, as comorbidities such as chronic obstructive pulmonary disease place them at high risk for complications of invasive diagnostic and staging procedures. Patients with possible lung cancer seem to be a population likely to benefit from an early multidisciplinary discussion to plan the biopsy strategy [4].

This study examines two groups of lung cancer patients distinguished by whether or not the biopsy strategy was planned with an early tumor board discussion. We retrospectively examined the impact that a multidisciplinary discussion had on the time interval from initial suspicion of lung cancer to completion of diagnosis, staging, and initiation of therapy. Because our focus was the efficient completion of the diagnostic and staging evaluation, our primary end-point was the completion of staging. Early discussion did not shorten this time interval, in fact, it seemed to be associated with delay. However, when the advanced stage cancer patients who were least likely to need chest-specific diagnostic and staging procedures were removed from the analysis, the delay was no longer statistically significant. Further dissection of the various time periods from imaging to therapy may improve the understanding of the process workflow. For example, we noticed that patients with stages I-III took 17 days longer after staging to commence therapy (Sx Tx). Since stage IV lung cancer is typically treated with systemic chemotherapy or palliation, it is less likely to be delayed by the involved planning required for preoperative evaluation or radiation therapy with other stages. However, waiting times for oncology visits, molecular testing, and radiation planning may still influence time delays. Other reasons for longer time intervals in the pre-treatment period might include timely access to endobronchial ultrasound, mediastinoscopy or interventional radiology; pulmonary function testing; cardiac evaluations; and quantitative lung perfusion nuclear testing; and so on. Establishing goals for the time from cancer suspicion to treatment initiation akin to door-to-balloon times in the field of cardiology may be part of future lung cancer care guidelines [9]. The American College of Surgeons does report cancer care quality measures relating to the time of lung cancer surgery and adjuvant chemotherapy of less than four months as of 2014 and an update is expected [10].

Data on times to diagnosis or therapy are more readily available in literature than time to staging. In evaluating the effect of tumor boards on time to treatment, Freeman et al. reported an average of 17 days from diagnosis to treatment with MTB discussion as compared to 29 days in a control group [2]. The ReCAP study showed a median of 33 days specifically in stage III lung cancer cases [5]. The Lehigh Valley Network uses single-day multidisciplinary clinic visits with different lung cancer specialists to encourage a team effort in the provision of high-quality cancer care. This group showed a trend toward a shorter time to therapy for stage III non-small cell lung cancer [11]. The timing of the tumor boards (before or after tissue diagnosis) is not explicit in the above data.

The limitations of our study include a selection bias that preferentially placed symptomatic, advanced-stage lung cancer patients into the control group with a disproportionate allocation of patients needing mediastinal staging to the case group (Table 1). We have accounted for such confounders while performing the regression analysis. Whether the need for mediastinal staging was driven by the early multidisciplinary discussion is not clear. Patients presenting with severe symptoms or radiographic concern for advanced cancer are more likely to be diagnosed rapidly, especially in the inpatient setting. The logistics of EBUS, mediastinoscopy, and interventional radiology scheduling can influence the time to completion of lung cancer staging. Stage migration may happen with delays in time from diagnosis to the institution of therapy and hence the need for efficiency. These data do not support the supposition that all lung cancer patients will benefit from early, formal tumor board discussions for planning tissue procurement. However, in complex situations, particularly in presumed early stages, this practice may be helpful. Certainly, a discussion between radiology and the physicians planning a non-radiologic biopsy, as mentioned in NCCN and American College of Chest Physicians (ACCP) guidelines, is prudent [3,12]. The added advantage of access to a palliative care physician early in the course of the disease that comes with an early tumor board discussion cannot be overstated.

## Conclusions

In summary, inter-departmental discussions may assist physicians in precise and personalized patient care in an arena that is increasing in complexity. Our study echoes guideline suggestions from Silvestri et al. (referenced above) that early discussion of biopsy strategies for diagnosis and staging is more likely to benefit patients who are candidates for stages II-III disease (groups B and C by ACCP criteria) than advanced lung cancer patient (group A) or early-stage patients (group D) who are operable and can go right to surgical resection. Our manuscript shares rare numerical data on timelines to important milestones in the process of cancer diagnosis and treatment. Using the above foundational data, perhaps future prospective studies may help to define the type of patients likely to derive a benefit from a pre-diagnosis tumor board. These quality metric data points will motivate primary referring providers to utilize the multidisciplinary team better and help refine the process workflow.
